# Supplementary material for: Interface-type tunable oxygen ion dynamics for physical reservoir computing
Source: Nat Commun. 2023 Nov 7;14:7176. doi: 10.1038/s41467-023-42993-x (PMC10630289; doi:10.1038/s41467-023-42993-x)
Supplement: Supplementary file 1 — Supplementary Information [file 41467_2023_42993_MOESM1_ESM.pdf]

Supplementary Information for

Interface-type tunable oxygen ion dynamics for physical reservoir computing

Zhuohui Liu[1,2#], Qinghua Zhang[1,3#], Donggang Xie[1#], Mingzhen Zhang[1,4], Xinyan Li[1,2], Hai Zhong[1,5], Ge Li[1,4], Meng He[1], Dashan Shang[6], Can Wang[1,4], Lin Gu[7], Guozhen Yang[1], Kuijuan Jin[1,4*], and Chen Ge[1,4*]

[1] Beijing National Laboratory for Condensed Matter Physics, Institute of Physics, Chinese Academy of Sciences, Beijing 100190, China

[2] College of Materials Science and Opto-Electronic Technology, University of Chinese Academy of
Sciences, Beijing 100049, China

[3] Yangtze River Delta Physics Research Center Co. Ltd., Liyang 213300, China

[4] School of Physical Sciences, University of Chinese Academy of Science, Beijing 100049, China

[5] School of Physics and optoelectronics Engineering, Ludong University, Yantai, 264025 Shandong, China

[6] Key Laboratory of Microelectronic Devices and Integrated Technology, Institute of Microelectronics, Chinese Academy of Sciences, Beijing 100029, China

[7] Beijing National Center for Electron Microscopy and Laboratory of Advanced Materials, Department of Materials Science and Engineering, Tsinghua University, Beijing 100084, China

[#] These authors contributed equally: Zhuohui Liu, Qinghua Zhang, Donggang Xie

* Correspondence and requests for materials should be addressed to K.J. (email: kjjin@iphy.ac.cn) or to C.G. (email: gechen@iphy.ac.cn).

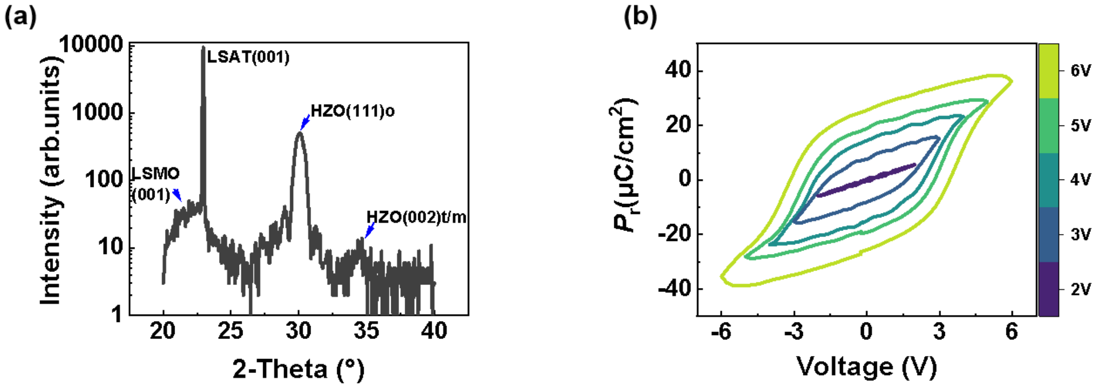

Supplementary Figure 1. Characteristics of LSAT/LSMO/HZO heterostructure. a X-ray diffraction of the heterostructure. **b** Polarization-voltage curve under different voltage pulses. The thickness of $La_{0.67}Sr_{0.33}MnO_3$ (LSMO) channel and $Hf_{0.5}Zr_{0.5}O_2$ (HZO) gate films were about 3.2 nm and 10 nm, respectively.

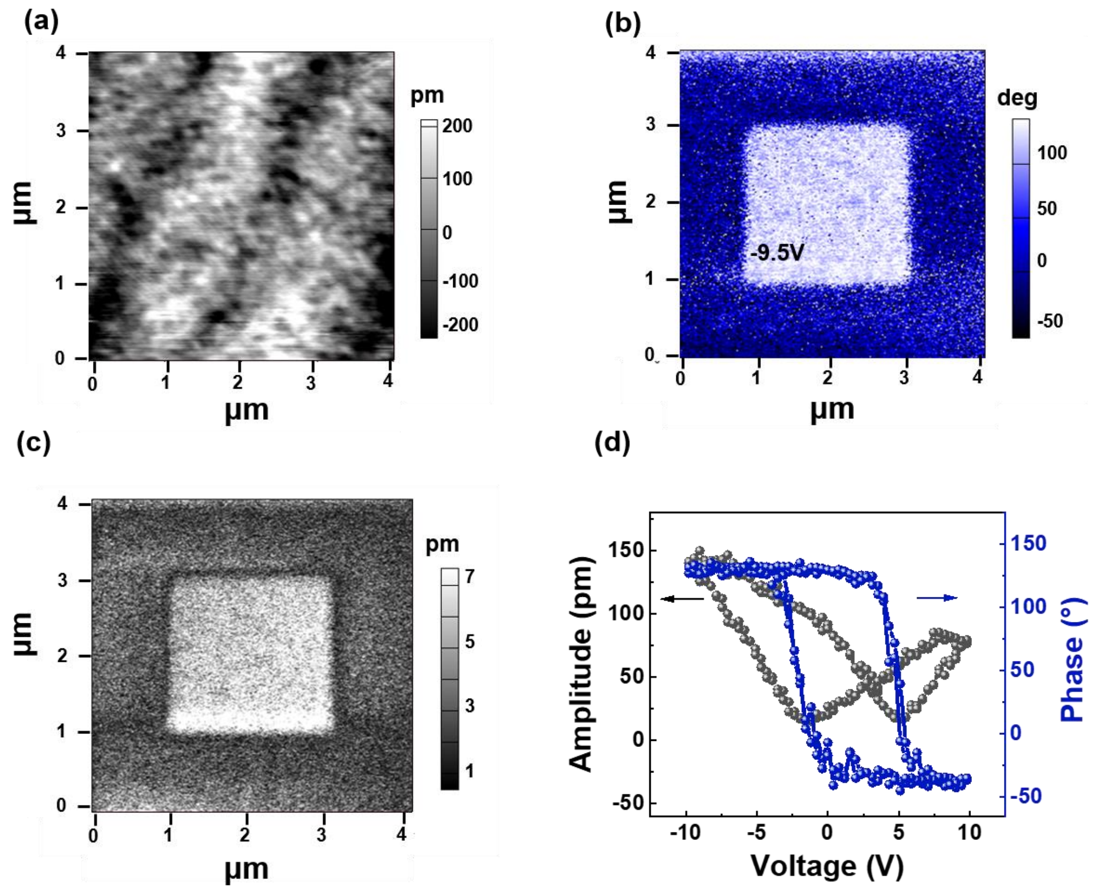

Supplementary Figure 2. Results of scanning probe microscope. a The surface topography of LSAT//LSMO/HZO. **b** PFM phase image and **c** amplitude of HZO films. **d** local PFM hysteresis curve. The 180° phase reversal and clear domain wall shows ferroelectric properties of HZO grown on LSMO/LSAT.

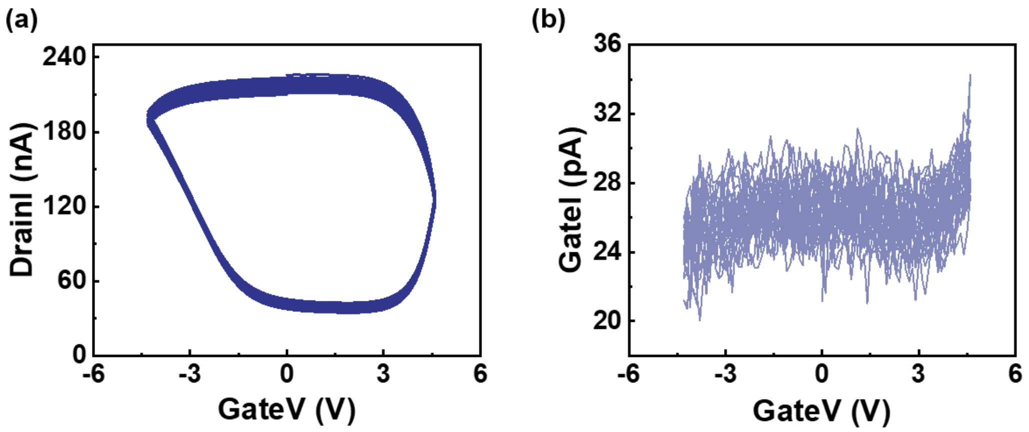

Supplementary Figure 3. Transfer curve of our FET device and corresponding leakage current. **a** By applying voltage to the HZO gate, the dynamic range of drain current reached 500 %. **b** Tiny leakage current was measured, thus we can exclude the current contribution from gate leakage.

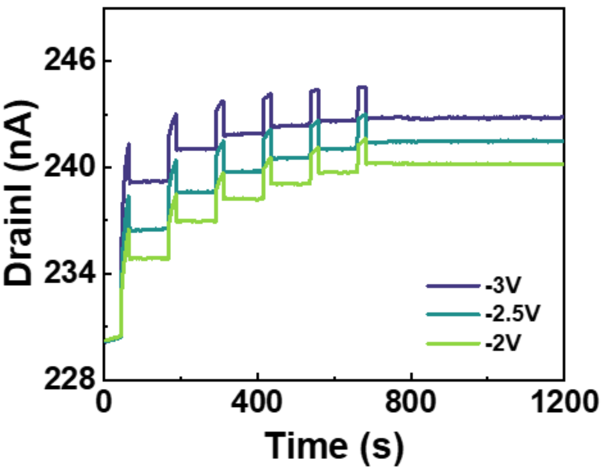

Supplementary Figure 4. Evolution of the drain current during different negative voltage stimulations.

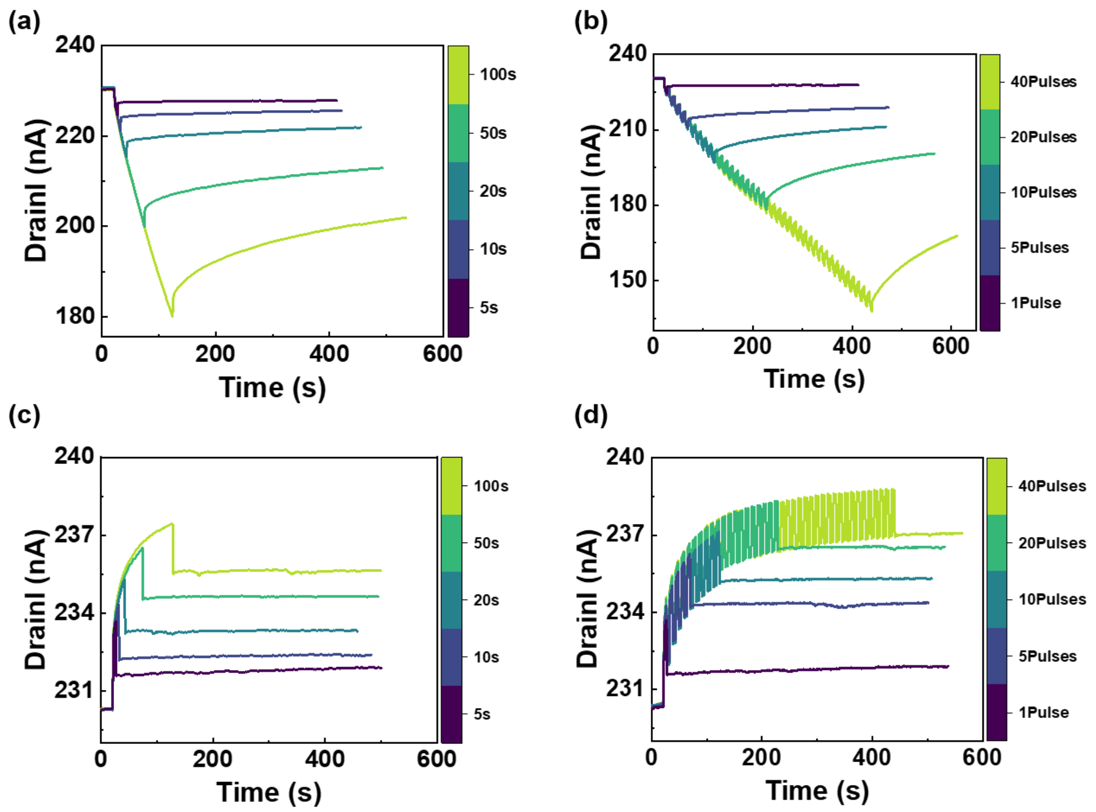

Supplementary Figure 5. The effect of width and quantity for voltage pulses. Drain current under various positive **a** pulse width and **b** pulse number, as well as negative **c** pulse width and **d** pulse number. When applying positive pulses, obvious relaxation process was observed, as a contrast, drain current modulated by negative pulses was non-volatile. The amplitude of positive and negative pulses are 3 V and -3 V, respectively. The width of each pulse is 5 s.

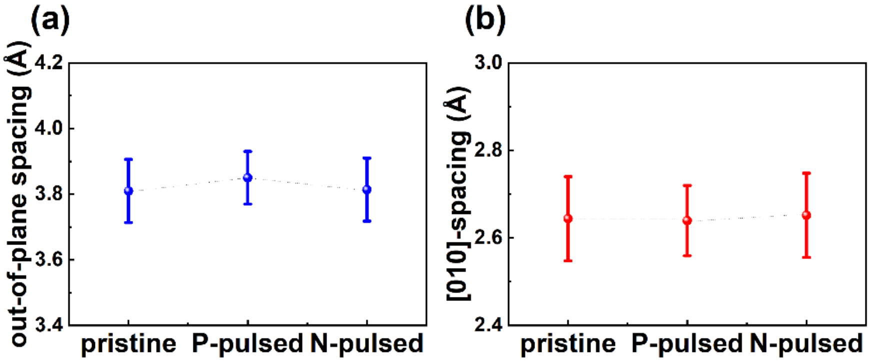

Supplementary Figure 6. Lattice analysis based on the STEM measurements. a Variation of out-of-plane lattice parameter in the pristine and pulsed LSMO films. **b** The in-plane spacing along [010] direction. The error bars represent computational error caused by the resolution limit of STEM image.

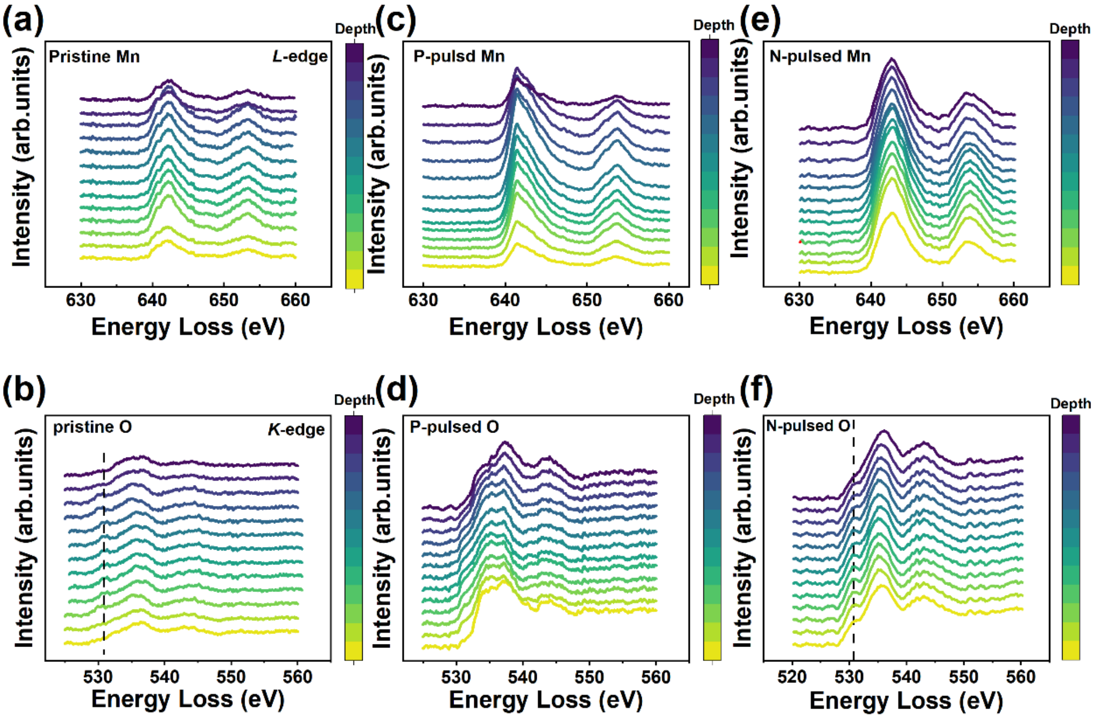

Supplementary Figure 7. Depth analysis of STEM-ELLS. Variation of the Mn *L*-edge from top to the bottom interfaces of pristine and pulsed LSMO films **a** pristine, **c** positively pulsed, **e** negatively pulsed. Variation of the O *K*-edge from top to the bottom interfaces of pristine and pulsed LSMO films. **b** pristine, **d** positively pulsed, **f** negatively pulsed.

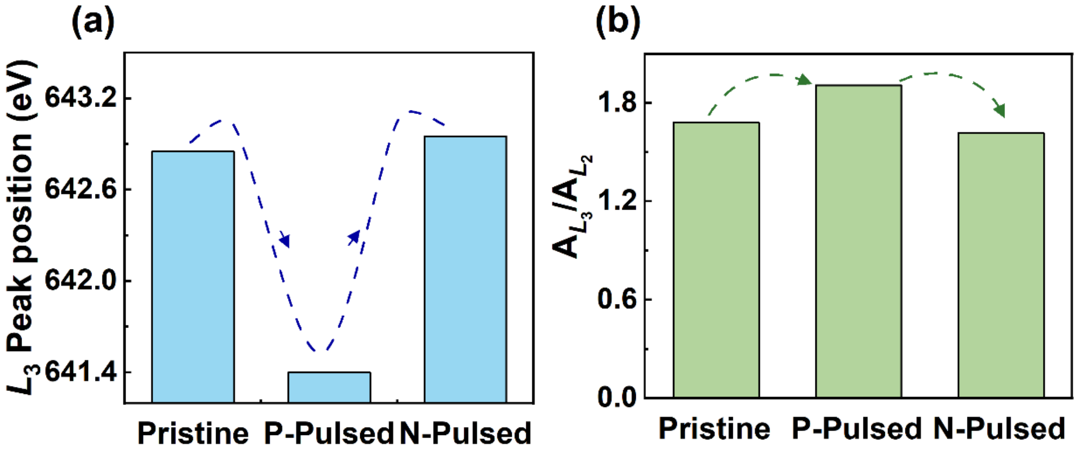

Supplementary Figure 8. Peak analysis of Mn *L*3 edge before and after the stimulation. a A significant shift towards lower energy after positive voltage stimulation indicates the reduction of Mn^{4+}. The shift of the peak towards higher energy after negative voltage stimulation confirms the oxidation of Mn^{3+}. **b** The ratio of peak area L_3/L_2 in the pristine and pulsed LSMO films.

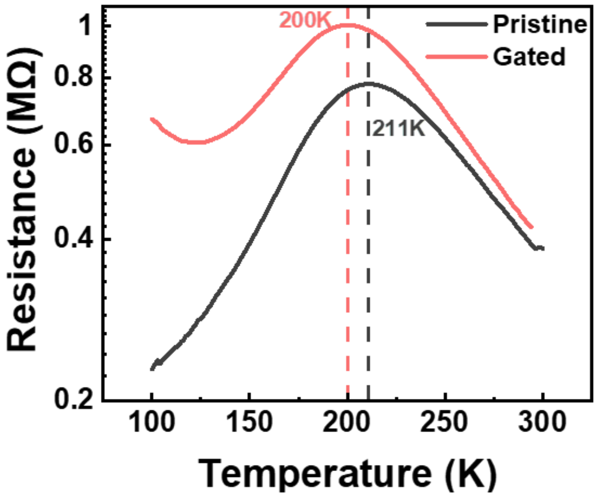

Supplementary Figure 9. Temperature-dependent resistance in the pristine and pulsed LSMO films. The resistance of LSMO was noticeably increased after the gating modulation.

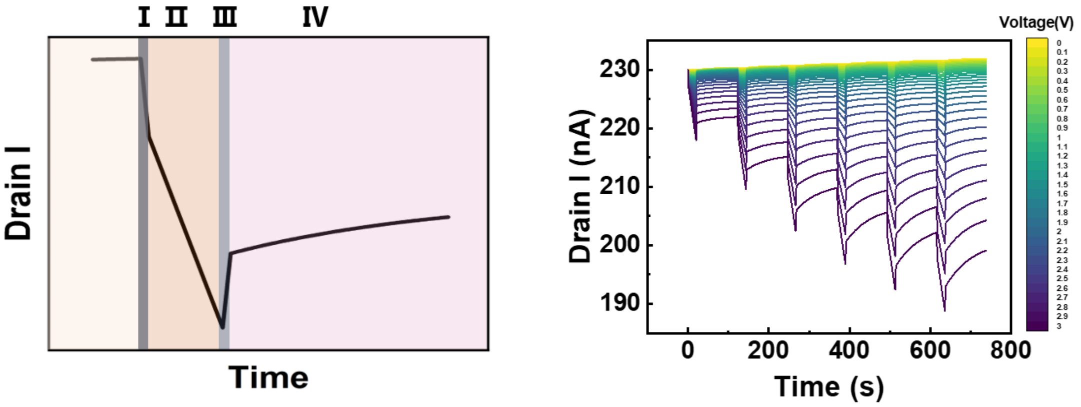

Supplementary Figure 10. The fitting current-voltage curve during the voltage stimulation.

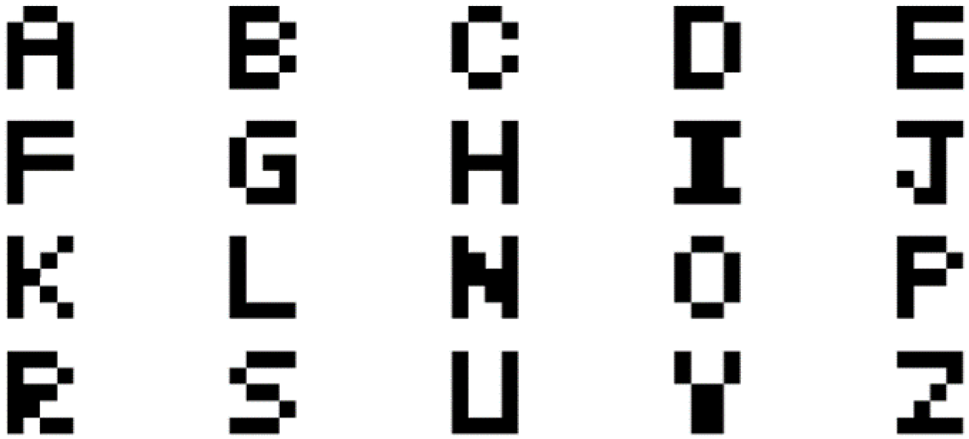

Supplementary Figure 11. All 20 kinds of digital letters used for the recognition task which generated using MatLab software.

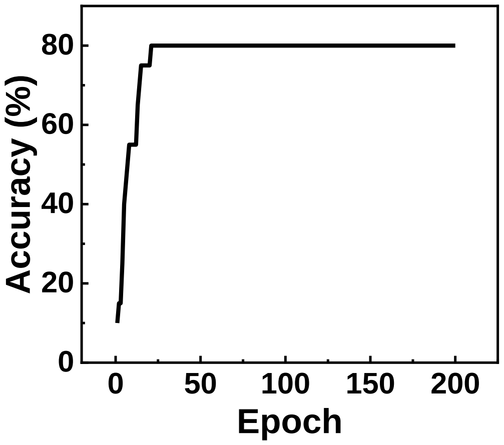

Supplementary Figure 12. Classification accuracy obtained through a linear model. Letters with the same number of “1” or “0” inputs in the same row cannot be distinguished by the linear model without processing by reservoir computing.

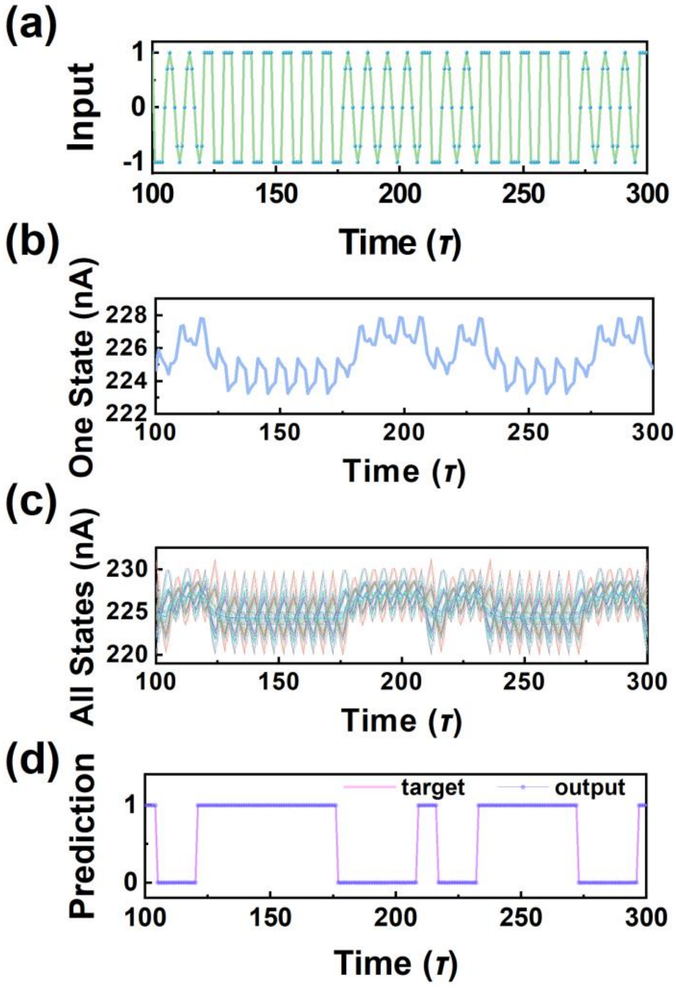

Supplementary Figure 13. Evolution of reservoir states during the waveform classification task. a Input waveform, which is transformed into the pulses through a mask process. **b** One typical virtual-node state and **c** all virtual-node states of the reservoir. **d** Classification results obtained from the reservoir system. The mask length of 50 demonstrated in the main text was the optimized parameter to get lowest error (4.044×10^{-8}). Here, we use the mask with length of 4 to schematically show the process of states' generation and output.

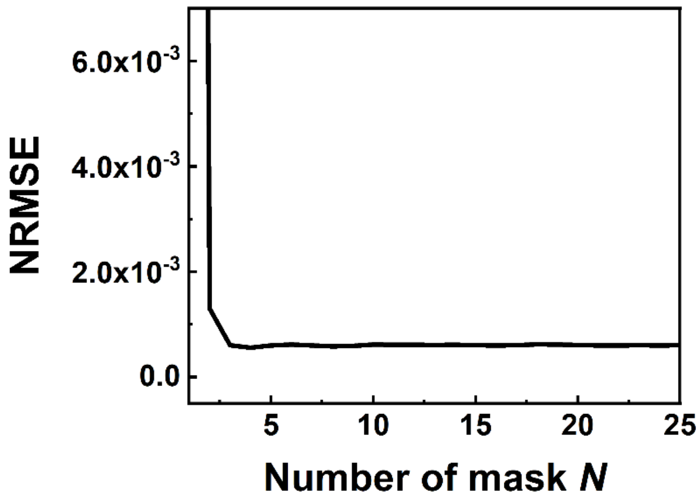

Supplementary Figure 14. The classification error (NRMSE) changes with the mask number.

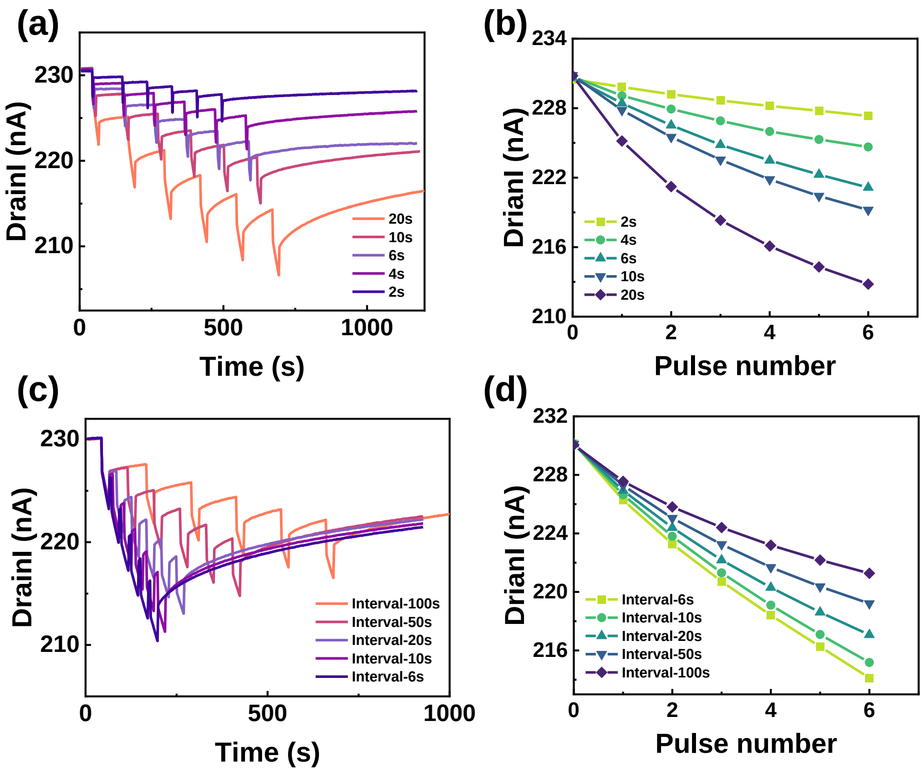

Supplementary Figure 15. Variation of the channel current under various positive pulse. **a** different pulse widths. **c** different intervals between each pulse. The amplitude of voltage is 2.5 V. **b**, **d** is extracted drain current data from **a** and **c**, respectively.

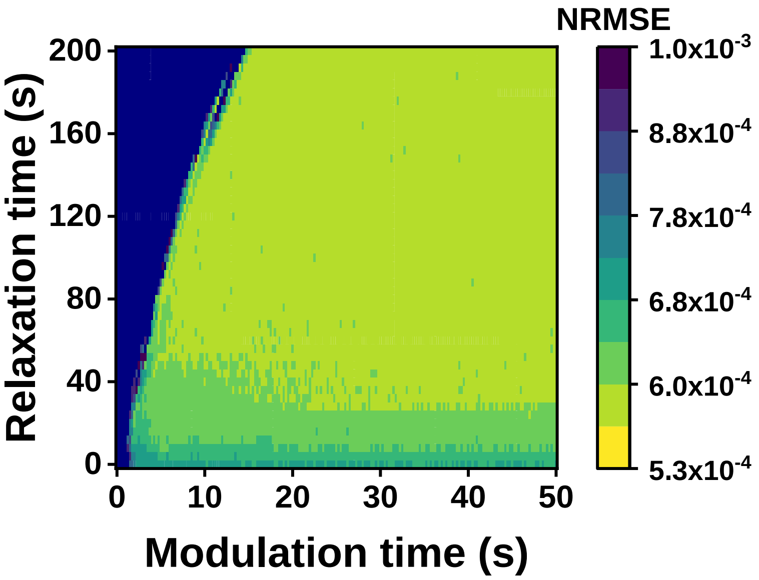

Supplementary Figure 16. NRMSE of Hénon map prediction as a function of pulse width and pulse interval.

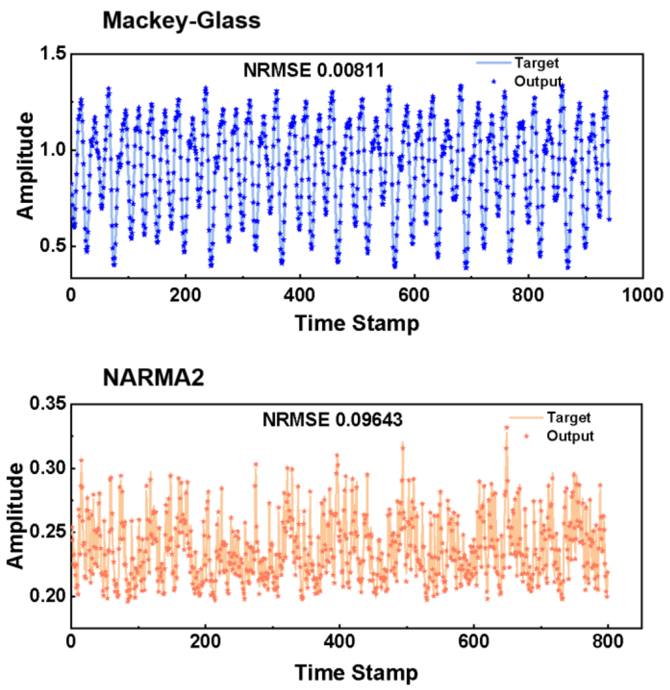

Supplementary Figure 17. The classification error (NRMSE) obtained in different time series prediction tasks. The NRMSE values of predicting Mackey-Glass oscillator[1] and NARMA2[2] tasks were 0.008 and 0.096, respectively.

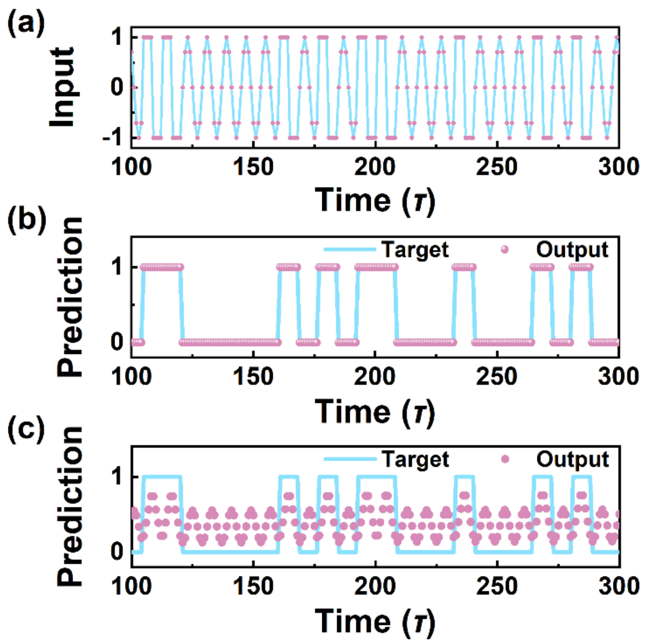

Supplementary Figure 18. Comparison of waveform prediction results between reservoir nonlinear model and linear model. **a** input waveform, **b** prediction result with PRC pre-processing, **c** prediction result with linear model.

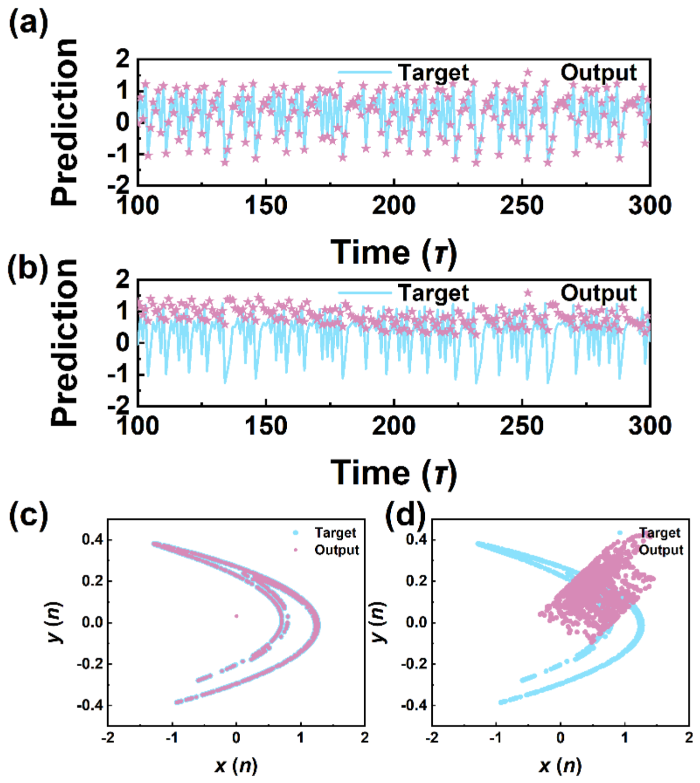

Supplementary Figure 19. Comparison of Hénon map prediction results between reservoir nonlinear model and linear models. a, **c** results obtained through nonlinear model, **b**, **d** results obtained through linear model.

Supplementary Table 1. The parameters used for simulation in Supplementary Figure 10.

Parameters	Values
k_1	-4.82412
k_2	0.01625
y_1	8.57408×10^{-4}
A_1	-8.51028×10^{-4}
b_1	0.47445
k_3	4.03075
y_2	-0.01151
A_2	0.01151
b_2	1.27601
A_3	9.40829
c_1	205.72315
b_3	7.73827
c_2	178.62049
k_4	-0.57756

Supplementary Table 2. Several reported reservoir systems and its performance in dealing with dynamic prediction compared with our work.

Devices	Tasks	NRMSE
Standard ESN system[3]	Hénon map prediction	0.091
Delay dynamic system[4]	Mackey – Glass oscillator	0.15
WO_x memristor[5]	second-order dynamic nonlinear system	0.0559
TaO_y dynamic memristor[6]	Hénon map prediction	0.046
α-In_2Se_3 based optoelectronic synapse[7]	MSO_5 task	0.105
Parallel eRNRS[8]	NARMA10 prediction	0.055
Ion-gating reservoir [9]	NARMA2 prediction	0.1414
HZO-based interface ion dynamic transistor (This work)	Hénon map prediction	5.75×10^{-4}
	Mackey – Glass oscillator	0.0081
	NARMA2 prediction	0.0964

Supplementary references

1. Jaeger H, Haas H. Harnessing Nonlinearity: Predicting Chaotic Systems and Saving Energy in Wireless Communication. *Science* **304**, 78-80 (2004).
2. Atiya AF, Parlos AG. New results on recurrent network training: unifying the algorithms and accelerating convergence. *IEEE transactions on neural networks* **11**, 697-709 (2000).
3. A. Rodan and P. Tino, "Minimum Complexity Echo State Network," in *IEEE Transactions on Neural Networks*, **22**(1)131-144, (2011).
4. Appeltant, L., Soriano, M., Van der Sande, G. *et al.* Information processing using a single dynamical node as complex system. *Nat Commun* **2**, 468 (2011).
5. Du, C., Cai, F., Zidan, M.A. *et al.* Reservoir computing using dynamic memristors for temporal information processing. *Nat Commun* **8**, 2204 (2017).
6. Zhong, Y., Tang, J., Li, X. *et al.* Dynamic memristor-based reservoir computing for high-efficiency temporal signal processing. *Nat Commun* **12**, 408 (2021).
7. Liu, K., Zhang, T., Dang, B. *et al.* An optoelectronic synapse based on α-In_2Se_3 with controllable temporal dynamics for multimode and multiscale reservoir computing. *Nat Electron* **5**, 761–773 (2022).
8. Liang, X., Zhong, Y., Tang, J. *et al.* Rotating neurons for all-analog implementation of cyclic reservoir computing. *Nat Commun* **13**, 1549 (2022).
9. Daiki Nishioka et al. Edge-of-chaos learning achieved by ion-electron–coupled dynamics in an ion-gating reservoir. *Sci. Adv*.**8**, eade1156 (2022).
